# Quantification of Retinal Vessel Myogenic Constriction in Response to Blood Pressure Peaks: Implications for Flicker Light-Induced Dilatation

**DOI:** 10.3389/fphys.2021.608985

**Published:** 2021-02-18

**Authors:** Lukas Streese, Anja Vaes, Denis Infanger, Ralf Roth, Henner Hanssen

**Affiliations:** Department of Sport, Exercise and Health, University of Basel, Basel, Switzerland

**Keywords:** retinal vessel analysis, baseline diameter, microvascular dysfunction, cardiovascular risk, endothelial function

## Abstract

**Background/Aims:**

Flicker-light induced retinal vessel dilatation (FID), a marker of microvascular endothelial function, has been shown to be blunted in sedentary cardiovascular risk patients (SR) as well as healthy physically active individuals (HA). This study aimed to quantify the retinal myogenic response to blood pressure (BP) peaks and its effects on consecutive FID for differentiation of microvascular health.

**Methods:**

Ten HA and eleven SR with a previously established restriction of arteriolar FID (aFID) (<2.2%) were invited in order to assess BP-induced myogenic constriction following a standardized handgrip task and a consecutive FID. BP was measured beat-to-beat.

**Results:**

The complete dataset of nine HA (3 female, mean age 65 years) and nine SR (5 female, mean age 61 years) individuals was analyzed. The central retinal arteriolar diameter equivalent (CRAE) was 183 ± 11 μm for HA and 176 ± 20 μm for SR. Initial baseline aFID was 1.6 ± 0.4% in HA and 1.6 ± 0.7% in SR. Systolic (*p* = 0.334) and diastolic (*p* = 0.245) BP increase following the handgrip task was in the range of 20–30% and comparable in both groups. BP increase was followed by a significantly higher arteriolar (−2.9 ± 1.3% vs. −1.3 ± 0.6%, *p* < 0.01) myogenic constriction in HA compared to SR. Moreover, in the consecutive assessment of FID directly after the BP-induced vessel constriction, aFID (4.1 ± 2.0% vs. 1.6 ± 0.9%, *p* < 0.01) was higher in HA compared to SR.

**Conclusion:**

Initial baseline aFID was blunted in HA and SR. Retinal myogenic constriction was impaired in SR compared to HA. The consecutive aFID after BP-induced myogenic constriction recovered in HA but remained blunted in SR. Additional assessment of retinal myogenic constriction needs to be considered to improve CV risk stratification and reduce false-positive findings of endothelial dysfunction in otherwise healthy active individuals.

**Clinical Trial Registration:**

ClinicalTrials.gov: NCT03986892 (https://clinicaltrials.gov/ct2/show/NCT03986892).

## Introduction

The retinal microcirculation is known as a reliable microvascular bed to quantify individual systemic cardiovascular (CV) risk (Wang et al., 2007; Flammer et al., 2013). Static retinal vessel analysis (SVA) non-invasively measures central retinal arteriolar (CRAE) and central retinal venular diameter equivalents (CRVE). Arteriolar narrowing and venular widening have been associated with stroke (Ikram et al., 2006), coronary heart disease (Wong et al., 2002) and higher CV mortality (Wang et al., 2007). In addition, venular widening has previously been associated with type 2 diabetes (Li et al., 2020). Retinal arteriolar and venular vessel diameters are predictive for long-term CV outcomes (McGeechan et al., 2009; Seidelmann et al., 2016) and the reclassification rate for the risk of stroke (McGeechan et al., 2009) and CV events (Seidelmann et al., 2016) is considered to be about 20%.

Dynamic retinal vessel analysis (DVA) non-invasively measures retinal microvascular endothelial function by quantifying arteriolar (aFID) and venular flicker light-induced vessel dilatation (vFID) over time. DVA measures shear stress-induced and nitric oxide (NO) mediated vascular dilatation. Dorner et al. (2003) showed that FID was significantly reduced after inhibition of NO synthase, supporting the assumption that NO is a key player in the regulation of retinal microvascular function. Therefore, decreased flicker light-induced dilatation (FID) reflects endothelial dysfunction (Heitmar and Summers, 2012; Machalinska et al., 2018) and is associated with CV risk and disease, such as aging (Kneser et al., 2009; Seshadri et al., 2016), obesity and diabetes (Kotliar et al., 2011; Patel et al., 2016; Sorensen et al., 2016), hypertension (Machalinska et al., 2018) and hypercholesterolemia (Nagele et al., 2018a) as well as heart failure (Nagele et al., 2018b). Gunthner et al. (2019) showed an all-cause mortality increase of 26% by every standard deviation decrease in vFID in end-stage renal disease patients.

Despite the available evidence that SVA and DVA are both promising techniques to reliably diagnose microvascular end-organ damage, we discussed the diagnostic challenge of using FID in specific populations in a previous study. In the EXAMIN AGE study, we reported that healthy and physically active individuals (HA) showed a blunted aFID comparable to sedentary CV risk patients (SR) (Streese et al., 2019). FID of both groups were comparable to previous studies investigating FID in CV risk patients (Seshadri et al., 2016; Nagele et al., 2018b). Therefore, we discussed this phenomenon of a blunted FID in HA as a physiological adaptation to exercise training. It represents a physiological dilatation of arterial baseline diameters in response to regular aerobic exercise training (Green et al., 2012). The predilated “exercised” artery lacks further dilatation capacity in response to flow-induced stimuli, for example resulting in a reduced flow-mediated dilatation (FMD) of the brachial artery (Celermajer et al., 1992; Green et al., 2012; Montero et al., 2013). We speculated that exercise-induced pre-dilatation of arterioles in the retinal microcirculation may lead to a reduced dilatation capacity, resulting in a blunted retinal FID. Our hypothesis was supported by our previous findings of higher arteriolar baseline diameters in HA compared to SR (Streese et al., 2019). A previous study under hypoxic conditions has also shown blunted aFID in predilated arteriolar vessels (Neumann et al., 2016).

This study aimed to differentiate between the blunted FID in HA and SR previously reported. We hypothesized that a BP-induced myogenic vasoconstriction prior to the standard DVA assessment would lead to a normalization of aFID in HA but not in SR.

## Materials and Methods

### Study Design

This cross-sectional study is an extension of the EXAMIN AGE study (Streese et al., 2018). Ten HA and eleven SR individuals with a previously blunted aFID (≤2.2%) were re-invited to investigate the myogenic constriction in response to BP increase and the consecutive FID immediately after the constriction stimulus. No cut-off values for FID have been defined yet. However, aFID of 2.3% or lower have previously been associated with higher CV risk (Al-Fiadh et al., 2014; Sorensen et al., 2016; Nagele et al., 2018b). Therefore, we re-invited HA and SR individuals who had an aFID ≤2.2% in their previous assessment within the EXAMIN AGE study. This study was conducted in accordance to the protocol and principles stated in the Helsinki Declaration (World-Medical-Association, 2013). The Ethics Committee of Northwest and Central Switzerland approved this study (EKNZ 2015-351). All participants signed a written informed consent prior the first measurement.

### Inclusion and Exclusion Criteria

Inclusion criteria have been described in detail previously (Streese et al., 2018, 2020b). Briefly, SR patients needed to have at least two CV risk factors, such as obesity, hypertension, diabetes, high triglyceride levels, high low-density lipoprotein levels (LDL), low high-density lipoprotein levels (HDL), or an active smoking status. HA individuals needed to be healthy without any of the CV risk factor described above. Additionally, SR needed to be sedentary [<3 metabolic equivalents (METs)/week] and HA needed to be physically active (>9 METs/week). Exclusion criteria were history of CV disease (only for HA), pulmonary or chronic inflammatory disease, macular degeneration, glaucoma, or any chronic eye disease.

### Retinal Vessel Analysis

We used the Dynamic Vessel Analyzer (DVA^®^; Imedos Systems GmbH, Jena, Germany) and a fundus camera (450FF, Carl Zeiss, Jena, Germany) to conduct the static and dynamic retinal vessel analysis, after pupil dilatation of one eye using tropicamide 0.5%.

#### Static Retinal Vessel Analysis

The standard procedures to conduct the SVA have been previously described in detail (Streese et al., 2018). Briefly, three images of the eye fundus, with an angle of 50°, were taken to calculate the CRAE, CRVE and the arteriolar-to-venular diameter ratio (AVR) using a standard analyzing software (Vesselmap 2^®^; IMEDOS Systems GmbH, Jena, Germany). Vessel diameters are presented in measuring units (mu). In the model of Gullstrand’s normal eye, 1 mu relates to 1 μm. The correlation coefficient and coefficient of variation for CRAE, CRVE, and AVR, using the described procedures ranged between 0.97–0.98 and 6.27–9.84% (*p* < 0.001 each) (Streese et al., 2020a).

#### Dynamic Retinal Vessel Analysis

**FIGURE 1:**
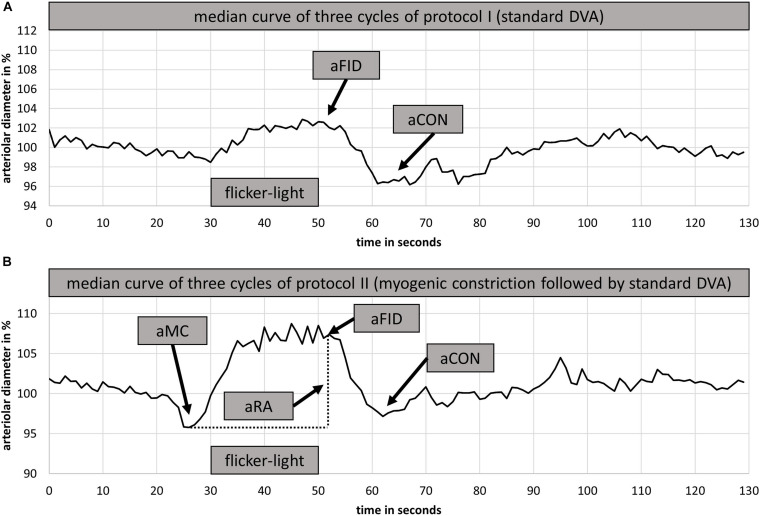
Examples of arteriolar median curves for DVA protocol I **(A)** and DVA protocol II **(B)**. DVA protocol I consisted of 50 s baseline recording followed by 20 s flicker light application and 80 s at rest **(A)**. DVA protocol II consisted of 50 s baseline recording, 40 s handgrip strength task at 30% 1RM, 30 s all-out handgrip strength task, 10 s at rest followed by 20 s of flicker light and a rest period of 80 s. DVA, dynamic retinal vessel analysis; aFID, maximal arteriolar flicker light-induced dilatation; aCON, maximal arteriolar vessel constriction after flicker stimulation; aMC, maximal arteriolar myogenic constriction before the flicker; aRA, maximal arteriolar range between arteriolar myogenic constriction and aFID; 1RM, one repetition maximum; BP, blood pressure.

Two DVA measurements were performed. The first DVA followed the standard method described in detail elsewhere (Streese et al., 2018, 2019). Briefly, the diameters of one arteriolar and one venular segment were marked and continuously measured over time. Three cycles with 20 s of high frequency flicker light were applied to non-invasively assess retinal endothelial function mediated by neurovascular coupling (Falsini et al., 2002; Polak et al., 2002). The individual baseline diameter, calculated from the first rest phase, was set as 100%. Vessel diameter changes were calculated in percentage-change based on the individual baseline value. The median curve of three identical cycles with 50 s baseline, 20 s flicker provocation and 80 s rest, was used to calculate aFID, vFID as well as the arteriolar (aCON) and venular constriction (vCON) after the flicker-light stimulus (Figure 1A). Maximum aFID and vFID were marked at the end of the flicker phase of the median curve, by avoiding to mark unphysiological peaks as shown in Figure 1B. aCON and vCON were marked between the end of the flicker phase and forty seconds after the flicker.

**FIGURE 2:**
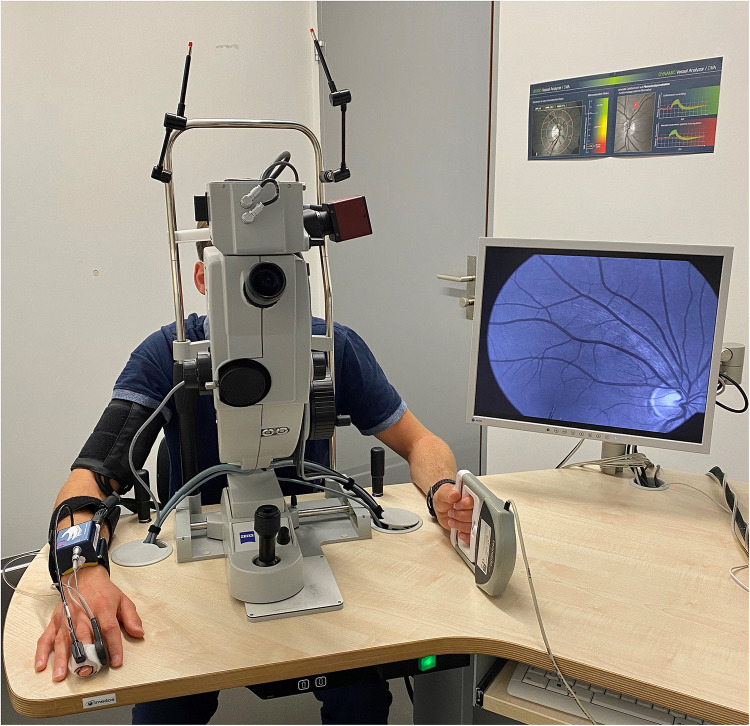
Setup for dynamic retinal vessel analysis (DVA) with the Dynamic Vessel Analyzer (DVA^®^; Imedos Systems GmbH, Jena, Germany) and a fundus camera (450FF, Carl Zeiss, Jena, Germany). Blood pressure was measured beat-to-beat on the middle finger of the right hand with the Finapres^®^ (Finapres Medical Systems B.V., Enschede, Netherlands). The Leonardo Mechanograph GF^®^ device (Novotec Medical GmbH, Pforzheim, Germany) on the left hand was used to increase the blood pressure of the participants. The produced power was continuously controlled using the Leonardo Mechanography BAS v4.4 software.

The second and consecutive DVA aimed to investigate the myogenic constriction as well as the FID directly after BP-induced vessel constriction. Participants needed to perform a standardized isometric handgrip task to elevate their BP, while the vessel diameters were continuously measured to calculate the arteriolar (aMC) and venular myogenic constriction (vMC). BP was measured beat-to-beat with the Finapres^®^ (Finapres Medical Systems B.V., Enschede, Netherlands). After a first baseline phase of 50 s, the participants were asked to press the Leonardo Mechanograph GF^®^ device (Novotec Medical GmbH, Pforzheim, Germany) for 40 s with 30% of their one repetition maximum (1RM), which was tested before with the same device. The produced power was continuously controlled using the Leonardo Mechanography BAS v4.4 software. An acoustic signal was installed to help the participants stay at 30% of their 1RM. A variance of 2% was tolerated. Participants needed to press the device as intensively as possible (all-out) for another 30 s to reach a maximum individual BP increase. During this high-intensity task, we continuously measured the retinal vessel diameters and the beat-to-beat BP response. The experimental set up is shown in Figure 2. Participants were verbally motivated by the investigator during the strength task. The 30 s all-out phase was followed by 10 s of rest, 20 s flicker light, to measure FID, and another 80 s rest phase, were the last 50 s were used to calculate the new baseline for the next cycle, before the next of three cycles started. We calculated the aMC, vMC, aFID, vFID, maximal arteriolar (aRA) and venular range between myogenic constriction and maximal FID (vRA), aCON, and vCON based on the median curve calculated from three identical cycles, comparable to protocol one. aFID, vFID, aCON, and vCON were marked identically to protocol one. aMC and vMC were marked during the 10 s rest phase before the flicker started (Figure 1B). The first 20 s in both DVA protocols were excluded as recommended by the manufacturer’s recommended protocol. At this point in time, the suggested protocol is not standard and our sample size is small. The reproducibility and variability of this new protocol need to be analyzed in larger sample sizes in the future to allow for better clinical interpretation. As an orientation, the correlation coefficient of the first two myogenic constriction and flicker cycles, with a rest phase of 80 s in between, was 0.96, 0.99, 0.98, and 0.71 for aMC, aFID, aRA, and aCON as well as 0.98, 0.92, 0.92, and 0.93 for vMC, vFID, vRA and vCON (*p* < 0.001 each). The coefficient of variation was −0.84, 0.88, 0.73, and −0.89 for aMC, aFID, aRA, and aCON as well as −0.63, 0.51, 0.33, and −1.18 for vMC, vFID, vRA, and vCON.

### Blood Pressure Assessment

BP was measured beat-to-beat during the whole measurement using a finger cuff with the Finapres^®^ device. The Finapres^®^ was calibrated by a BP measurement at the upper arm and a sensor measuring the altitude difference between the finger cuff and the heart. BP in rest was calculated by taking the mean of 10 s after 10 min of rest in a quiet room in a sitting position. BP differences were reported as means as well as in percentage with respect to the second DVA protocol. Baseline BP was calculated by averaging systolic and diastolic BP 10 s before the exercise task started. Maximum BP was calculated by averaging 10 s at the end of the all-out exercise phase.

### Statistical Analysis

Population characteristics are reported in mean and standard deviation (SD). Retinal vessel diameter changes are presented in percentage, based on the individual baseline. Group differences were calculated with *t*-tests, separately calculated for each parameter. Paired sample *t*-tests were used to calculate aFID, vFID, aCON, and vCON differences between DVA protocol I and II for each group separately. We used boxplots and histograms to test the symmetry of the value distribution. All statistics were performed with a two-sided significance level of 0.05. Figures that represent curve progressions are median curves of one representative participant. Therefore, DVA values can differ from Tables (mean values of all participants) and Figures. The statistic program R (Version 3.5.0) was used to calculate the statistics, R and Excel 2016 was used to generate graphs.

## Results

We re-invited 10 HA and 11 SR participants from the previous EXAMIN AGE study with an aFID ≤ 2.2%. One participant was excluded because of use of eye drops to reduce the intraocular pressure (IOP). Two participants were excluded based on insufficient data quality. Population characteristics of the final dataset, including nine HA and nine SR, are described in Table 1. Groups did not significantly differ in age, height, systolic and diastolic BP as well as IOP. SR were characterized by higher weight, BMI, glucose and triglyceride levels as well as lower HDL levels and cardiorespiratory fitness (CRF). Six SR patients had a high BMI, two had hypertension, three were active smokers, five had high glucose levels, five had high triglyceride levels, four had low HDL levels, and one patient had high LDL levels. All patients had at least two of the above described CV risk factors.

**TABLE 1:** Population characteristics.

	**HA (*n* = 9) mean ± SD**	**SR (*n* = 9) mean ± SD**	***p***
Sex (f/m)	3/6	5/4	
Age (years)	65 ± 9	61 ± 6	0.352
Height (cm)	168 ± 8	168 ± 11	0.885
Weight (kg)	67 ± 7	91 ± 13	<0.001
BMI (kg/m^2^)	23.5 ± 1.7	32.5 ± 3.8	<0.001
Systolic BP (mmHg)	127 ± 9	138 ± 19	0.156
Diastolic BP (mmHg)	77 ± 5	82 ± 9	0.212
Glucose (mmol/l)	4.9 ± 0.52	5.7 ± 1.1	0.047
Triglyceride (mmol/l)	0.97 ± 0.17	1.67 ± 0.33	<0.001
HDL (mmol/l)	1.94 ± 0.49	1.25 ± 0.32	0.006
LDL (mmol/l)	2.89 ± 0.64	3.65 ± 1.01	0.081
VO_2_ peak (ml/min/kg)	40 ± 11	26 ± 3	0.004
IOP (mmHg)	15 ± 3	14 ± 2	0.378
CRAE (μm)	183 ± 11	176 ± 20	0.422
CRVE (μm)	206 ± 11	213 ± 20	0.429
AVR	0.89 ± 0.06	0.83 ± 0.08	0.079
Arteriolar vessel segment (μm)	116 ± 8	107 ± 12	0.094
Venular vessel segment (μm)	139 ± 22	141 ± 14	0.836

*HA, healthy active; SR, sedentary at risk; SD, standard deviation; p, t-test level of significance for group differences; BMI, body mass index; BP, blood pressure; HDL, high-density lipoprotein; LDL, low-density lipoprotein; VO_2_ peak, peak oxygen uptake; IOP, intraocular pressure; CRAE, central retinal arteriolar diameter equivalents; CRVE, central retinal venular diameter equivalents; AVR, arteriolar-to-venular diameter ratio.*

### Microvascular Phenotype

**FIGURE 3:**
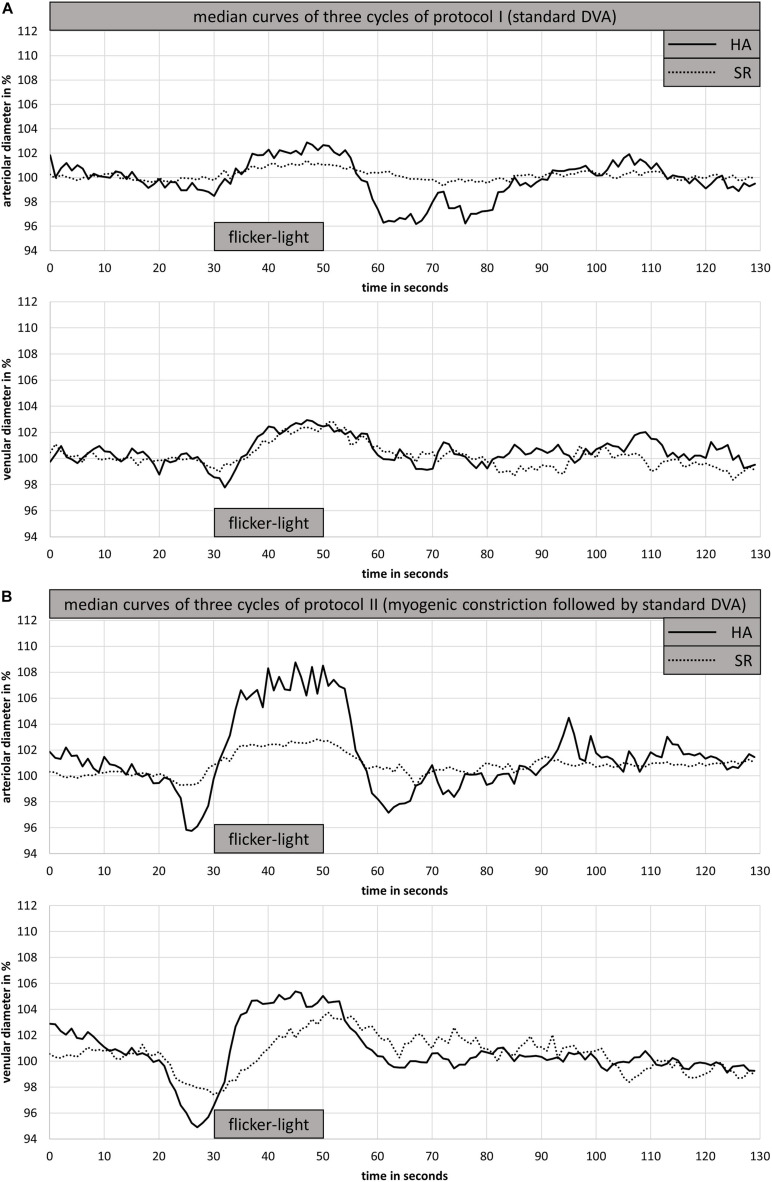
Arteriolar and venular median curves for DVA protocol I **(A)** and DVA protocol II **(B)** of one representative HA and SR participant. DVA protocol I consisted of 50 s baseline recording followed by 20 s flicker light application and 80 s at rest **(A)**. DVA protocol II consisted of 50 s baseline recording, 40 s handgrip strength task at 30% 1RM, 30 s all-out handgrip strength task, 10 s at rest followed by 20 s of flicker light and a rest period of 80 s. DVA; dynamic retinal vessel analysis; HA, healthy active; SR, sedentary patients with increased cardiovascular risk.

HA had wider CRAE (183 ± 11 μm vs. 176 ± 20 μm, *p* = 0.422) and narrower CRVE (206 ± 11 μm vs. 213 ± 20 μm, *p* = 0.429) as well as a higher AVR (0.89 ± 0.06 vs. 0.83 ± 0.08, *p* = 0.079) compared to SR. In addition, the investigated arteriolar vessel segment was wider in HA compared to SR (116 ± 8 μm vs. 107 ± 12 μm, *p* = 0.094). However, these differences were not statistically significant. HA and SR showed a comparable blunted aFID and vFID in protocol I (standard DVA protocol). Also, aCON and vCON did not significantly differ between groups (Figure 3A and Table 2).

**TABLE 2:** Mean and standard deviation of vascular response on dynamic retinal vessel analysis in percentage.

	**Protocol I (standard DVA)**				**Protocol II (myogenic constriction followed by standard DVA)**							
	**aFID**	**aCON**	**vFID**	**vCON**	**aMC**	**aFID**	**aRA**	**aCON**	**vMC**	**vFID**	**vRA**	**vCON**
HA (*n* = 9)	1.6 ± 0.4	−1.1 ± 1.0	4.5 ± 2.7	−0.5 ± 0.5	−2.9 ± 1.3	4.1 ± 2.0*	7.0 ± 2.9	−1.7 ± 1.7	−3.1 ± 1.4	4.7 ± 2.2	7.8 ± 1.9	−1.1 ± 1.1
SR (*n* = 9)	1.6 ± 0.7	−1.1 ± 1.0	3.6 ± 1.4	−1.2 ± 1.0	−1.3 ± 0.6	1.6 ± 0.9	3.0 ± 1.1	−1.3 ± 0.9	−1.6 ± 0.6	3.0 ± 1.1	4.6 ± 1.3	−0.9 ± 0.8
*p*	0.887	0.973	0.425	0.125	0.006	0.005	0.002	0.464	0.017	0.057	0.001	0.710

DVA, dynamic retinal vessel analysis; HA, healthy active; SR, sedentary at risk; p, t-test level of significance for group differences; aFID, arteriolar flicker light induced vessel dilatation; aCON, arteriolar vessel constriction after flicker stimulation; vFID, venular flicker light induced vessel dilatation; vCON, venular constriction after flicker stimulation; aMC, arteriolar myogenic constriction; aRA, maximal arteriolar range between arteriolar myogenic constriction and aFID; vMC, venular myogenic constriction; vRA, maximal venular range between venular myogenic constriction and vFID.

*Statistically significant difference between DVA protocol I and II.

**FIGURE 4:**
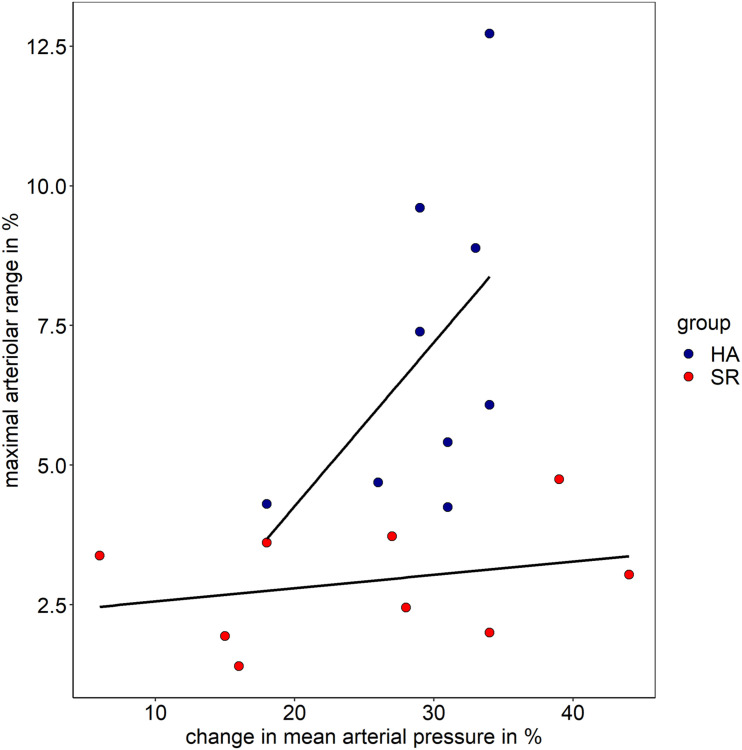
Association of maximal arteriolar range and change in mean arteriolar pressure in healthy active individuals (HA) and sedentary cardiovascular risk patients (SR).

Both groups showed comparable BP increase following the handgrip task in protocol II (myogenic constriction followed by standard DVA). The mean systolic BP raised in HA and SR from 130 ± 6 mmHg and 137 ± 17 mmHg to 173 ± 4 mmHg and 176 ± 8 mmHg. The mean diastolic BP in HA and SR raised from 78 ± 4 mmHg and 81 ± 8 mmHg to 120 ± 6 mmHg and 114 ± 15 mmHg. The mean BP increase was 25 ± 3% and 22 ± 9% systolic (*p* = 0.334) as well as 35 ± 4% and 29 ± 17% diastolic (*p* = 0.245) for HA and SR, respectively. The arteriolar (aMC: −2.9 ± 1.3% vs. −1.3 ± 0.6%, *p* < 0.01) and venular (vMC: −3.1 ± 1.4% vs. −1.6 ± 0.6%, *p* < 0.05) BP-induced myogenic vessel constriction was higher in HA compared to SR (Figure 3B and Table 2). The following mean aFID was higher in HA (4.1 ± 2.0%) compared to SR (1.6 ± 0.9, *p* < 0.01). vFID did not significantly differ between the groups (Figure 3B and Table 2). aRA, defined as maximal range of arteriolar reactivity from maximal myogenic constriction to maximal dilatation, was higher in HA (7.0 ± 2.9%) compared to SR (3.0 ± 1.1%, *p* < 0.01). vRA was also higher in HA (7.8 ± 1.9%) compared to SR (4.6 ± 1.3%, *p* < 0.001). Vessel constriction after the flicker did not significantly differ between the groups (Table 2). Higher BP increase during protocol II was associated with higher aRA in both groups, with a stronger association in HA compared to SR (Figure 4).

HA showed a 2.5% higher aFID in DVA protocol II (4.1%) compared to protocol I (1.6%, *p* < 0.01). SR showed no significant differences in aFID between the DVA protocols. No significant differences in vFID, aCON, and vCON between the DVA protocols were observed in both groups (Table 2).

## Discussion

Microvascular arteriolar function is of utmost importance to regulate constant blood flow and oxygen delivery to maintain organ function. Arteriolar function is often referred to mainly as endothelial function, which represents vessel dilatation in response to nitric oxide or other vessel dilators as a means of local autoregulation, classically in response to shear stress stimuli. Blood pressure-induced constriction is part of the vascular autoregulation and is responsible for constant blood flow during a range of arteriolar blood pressure changes. The so called “*Bayliss effect*” leads to a contraction of smooth muscle cells when arterial or transmural pressure rises, resulting in myogenic constriction (Pournaras et al., 2008). The underlying mechanisms have been extensively discussed elsewhere (Barabas et al., 2020). Retinal myogenic constriction has scarcely been studied to date.

Our results demonstrate that the unilateral view on retinal arteriolar function by measuring solely retinal endothelial function, limits the diagnostic accuracy of assessing microvascular function. HA and SR, as defined in our study, are characterized by different CV risk profiles and physical activity behavior, yet both showed a comparable blunted aFID. The mean aFID in both groups was 1.6%. We hypothesized that HA had predilated arterioles as a physiological adaptation to regular exercise training, which lead to a reduced dilatation capacity and, therefore, to a reduced FID. The individual vessel diameters show a high inter-individual variability depending on the magnification factor and the anatomy of each participant. This limits the comparability of single vessel diameters between participants. Nonetheless, the measured arteriolar segment and the summarized arteriolar calibers (CRAE) were higher in HA compared to SR but failed statistically significance. To reduce the high inter-individual variability, we would like to recommend use of AVR to estimate the relative state of dilatation of arteries and venules. The AVR was also higher in HA compared to SR but not statistically significant different. However, the results of our second DVA protocol, with an integrated measurement of myogenic constriction, showed that isometric handgrip exercise lead to a similar BP increase in HA and SR but resulted in a different microvascular response. HA showed higher BP-induced vessel constriction (aMC) compared to SR, followed by a mean aFID of 4.1% in the consecutive assessment. BP-induced vasoconstriction resulted in a lower baseline diameter and a higher dilatation capacity leading to a 2.5% higher aFID in HA compared to SR. SR only showed a BP-induced vasoconstriction of −1.3%, which did not result in a higher consecutive aFID. We would like to speculate that BP-induced vasoconstriction mitigated predilatation of arterioles in HA resulting in a higher aFID. However, it is also possible that SR already had pre-constricted arterioles due to functional narrowing based on a higher CV risk profile, which resulted in a reduced constriction response. Nevertheless, reduced baseline diameters by myogenic constriction lead to a significant higher aFID in HA but not in SR. Our hypothesis needs to be validated in future studies, for example by use of *in vitro* experiments. Such basic science-related approaches would allow for an in depth investigation of underlying structural and functional mechanisms, such as verification of dilatation capacity and vascular wall remodeling, NO reactivity in combination with oxidative stress or other vasoactive substances. We previously showed that NO bioavailability (Hanssen et al., 2011) as well as oxidative stress (Streese et al., 2020a,b) were associated with retinal vessel diameters. However, the dilatation response in the microcirculation is triggered by a balance of multiple mechanisms, such as endothelin (Kuchan and Frangos, 1993), prostaglandins (Koller et al., 1993), acetylcholine (Martin et al., 1996), or smooth muscle hyperpolarization (Busse et al., 2002). More research is needed to investigate these and further mechanisms in the retinal microcirculation to better understand the underling mechanism of a blunted FID. First and foremost, future studies will have to define normative data and cut-off values to differentiate variance of normality from pathology.

Previous studies reported an aFID of 3.6% for healthy controls, 2.3% for patients with CV risk factors and 0.9% for heart failure patients (Nagele et al., 2018b). Comparing these results with the 2.5% higher aFID in HA compared to SR after BP-induced vasoconstriction strengthens the clinical relevance of measuring the myogenic response in individuals with blunted aFID. We recommend our protocol for use in clinical practice in order to reduce false positive diagnosis of endothelial dysfunction in individuals who may have reason for pre-dilatation, such as physically active individuals. The phenomenon of blunted endothelial function in physically active individuals has previously been reported by different studies investigating endothelial function with FMD in the brachial artery (Green et al., 2013; Montero et al., 2013). The resting diameter seems to play a decisive role in FMD measurements, because higher resting diameters lead to a lower FMD response (Celermajer et al., 1992; Rembold et al., 2003).

As shown in the descriptive Figure 4, the amount of BP increase is positively associated with the maximal range of arteriolar reactivity in healthy individuals. In CV risk patients, the range of microvascular reactivity is blunted, irrespectively of the increase in BP (protocol II). We therefore recommend use of a standardized handgrip task aiming at maximal BP increase to provoke maximal microvascular reactivity and optimize differentiation of responder (high vascular reactivity) from non-responder (blunted vascular reactivity).

Venular myogenic constriction is also impaired in SR compared to HA. However, the BP stimulus did not lead to an increase in the consecutive vFID as compared to standard DVA protocol I.

Homeostatic arteriolar constriction is essential in protecting the capillaries from high pressure peaks. A loss of myogenic constriction capacity may lead to capillary damage and potentially to hemorrhages (Barabas et al., 2020). Diabetic patients with a diminished myogenic response have been reported to develop diabetic retinopathy earlier compared to diabetic patients with normal myogenic response (Tecilazich et al., 2016). Nussbaumer et al. (2014) suggested a reduced myogenic vasoconstriction of retinal arteriolar diameters in response to an acute exercise stressor in seniors compared to young adults as an age-related loss of myogenic constriction capacity.

**FIGURE 5:**
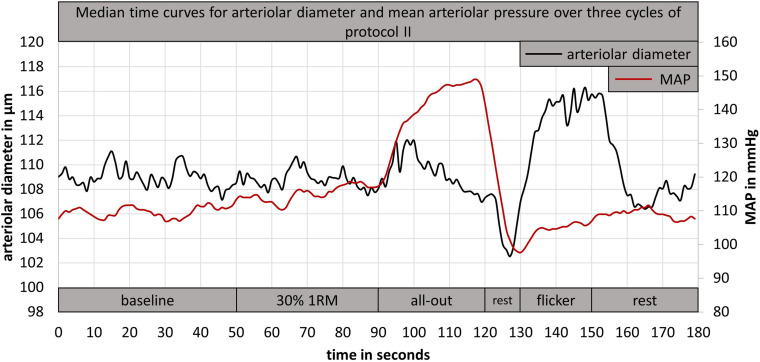
Example for a median time curve of arteriolar diameter and mean arteriolar pressure, calculated from three cycles of DVA protocol II of one participant. DVA; dynamic retinal vessel analysis; MAP, mean arteriolar pressure; 1RM, one repetition maximum.

Previous studies that investigated retinal vessel myogenic constriction did not standardize the exercise task that was induced to raise the blood pressure. Blum et al. (2005) used a 1.5 kg weight, which had to be held upright for 3 min in the right hand. Participants in the study from Jeppesen et al. (2007) had to lift a 2.4 kg weight for 3 min with the right arm. Holding or lifting a defined weight without adaptation to individual power capacity, limits the comparability between participants. Therefore, we implemented a standardized isometric handgrip task to elevate BP and investigate retinal myogenic constriction while vessel diameters were continuously measured (DVA protocol II). The first handgrip task of 30% 1RM for 40 s led to a small BP increase without a significant vessel response (Figure 5). Nearly all participants showed a strong BP increase during the following all-out handgrip task, which induced a time-delayed exaggerated myogenic constriction (Figure 5). We implemented a short rest phase of 10 s after the all-out phase. During this phase, MAP showed a fast recovery while the arteriolar vessel diameters further decreased (Figure 5). We therefore recommend implementation of such a short rest phase after a standardized handgrip task, taking into account the time delay between BP increase and corresponding myogenic constriction.

The direct effect of increased systemic MAP, during DVA protocol II, on the IOP and the ocular perfusion pressure was not investigated. Measuring the IOP simultaneously during the DVA was not possible. The Egna–Neumarkt Glaucoma Study showed a strong correlation (*r* = 0.94, *p* < 0.001) between systemic BP and IOP (Bonomi et al., 2000). However, the BP-induced IOP elevation seems to be moderate. Ten mmHG systolic and diastolic BP increase resulted in an IOP elevation of 0.20–0.44 mmHg and 0.40–0.85 mmHg, comparing different follow-up studies (Costa et al., 2009). A fast rise of systemic BP might induce higher choroidal volume and induce an increase in IOP. Further research is needed to investigate the direct effect of elevated systemic BP on IOP and implications for retinal vessel reactivity.

### Limitations

Our study was small in size with a focus on the principles of applied vascular physiology and definition of a new methodological technique. It investigated a population of highly active and healthy older individuals as well as older patients with increased CV risk. This study was conducted to better understand the vascular physiology in CV risk patients. Unfortunately, the sample size was too small to conduct a subgroup analysis to differentiate the vessel response between patients with or without hypertension or obesity. Therefore, these results would need to be confirmed in a larger cohort of patients with a distinct CV risk profile. Therefore, these results cannot be transferred to other populations. However, we were able to show that investigating the retinal myogenic response may be an essential addition to standard DVA in order to correctly evaluate endothelial function. Unfortunately, measuring the IOP during the dynamic retinal analysis to calculate ocular perfusion pressure and to better understand the local retinal pressure conditions was not possible. The reproducibility of DVA protocol II was assessed by comparing the first two myogenic constriction and flicker cycles of all participants, showing a good reproducibility by comparing two cycles in one assessment with 80 s recovery in between. However, the reproducibility and day-to-day variability of DVA protocol II needs to be investigated in future studies. Our results represent a first proof of principle approach asking for implementation in larger scale prospective clinical studies that need to define normal and cut-off values in healthy and normal active individuals, which was beyond the scope of our study.

## Conclusion

This study, for the first time, presents a new method to apply routine assessment of retinal myogenic constriction in health and disease. As hypothesized, BP-induced myogenic vasoconstriction prior to a standard assessment of FID lead to a normalization of aFID in HA but not in SR. Our protocol can differentiate between CV risk patients and physically active healthy individuals by inducing myogenic constriction prior to the flicker stimulus. Depending on the individual medical history as well as the level of physical activity and fitness, we recommend incorporation of myogenic constriction assessments to standard retinal vessel analysis as a microvascular biomarker for CV risk. Additional assessment of retinal myogenic constriction may improve individual CV risk stratification by avoiding false-positive diagnosis of endothelial dysfunction in otherwise healthy individuals. Whether retinal myogenic constriction by itself has additional predictive value for the development of CV disease has to be elucidated in future prospective clinical studies.

## Data Availability Statement

The raw data supporting the conclusions of this article will be made available by the authors, without undue reservation, to any qualified researcher.

## Ethics Statement

The studies involving human participants were reviewed and approved by the Ethics Committee of Northwest and Central Switzerland. The patients/participants provided their written informed consent to participate in this study.

## Author Contributions

LS designed the study, helped to perform the study, and wrote the first draft of the manuscript. AV mainly performed the study. DI gave his statistical input and revised the manuscript. RR gave his methodical input and critically revised the manuscript. HH designed the study, critically discussed the results, and revised the manuscript. All authors read and approved the final version of the manuscript.

## Conflict of Interest

The authors declare that the research was conducted in the absence of any commercial or financial relationships that could be construed as a potential conflict of interest.

## Abbreviations

aCON: arteriolar vessel constriction after flicker stimulation
aFID: arteriolar flicker light induced vessel dilatation
aRA: maximal arteriolar range between arteriolar myogenic constriction and aFID
aMC: arteriolar myogenic constriction
DVA: dynamic retinal vessel analysis
HA: healthy active
SR: sedentary at risk
vCON: venular constriction after flicker stimulation
vFID: venular flicker light induced vessel dilatation
vRA: maximal venular range between venular myogenic constriction and vFID
vMC: venular myogenic constriction.
